# Manifestation of Trauma: The Effect of Early Traumatic Experiences and Adult Attachment on Parental Reflective Functioning

**DOI:** 10.3389/fpsyg.2017.00449

**Published:** 2017-03-24

**Authors:** Pamela San Cristobal, Maria P. Santelices, Daniel A. Miranda Fuenzalida

**Affiliations:** Psychology, Pontificia Universidad CatolicaSantiago, Chile

**Keywords:** childhood trauma, adult attachment, parental reflective functioning, prementalization

## Abstract

There are many risk factors that make the transition to parenthood difficult, even in the best of circumstances. One such risk factor is the experience of parental childhood trauma, which has the potential to affect the parent/child relationship, both in terms of attachment style parental reflective functioning. This study aims to expand on the line of research concerned with the effects that trauma has once that child transitions into adulthood and into parenthood by looking at the role that the experience of trauma and adult attachment has in relation to parental reflective functioning. This study assessed mothers (*N* = 125) by using the CTQ (childhood experience of trauma), ECR (adult attachment), and the PRFQ (parental RF). Our study found that in the presence of physical neglect, insecure attachment had a particularly deleterious effect on maternal reflective functioning. This relationship was not as strong in the absence of physical neglect.

## Early experiences of trauma and the attachment system

The experience of trauma during childhood has been the subject of many empirical studies and its effects well documented, affecting different domains of mental health, and general wellbeing (Lieberman and Van Horn, 2008; Spratt et al., 2012). Consequently, clinical and empirical interest continues to drive research on the effects of trauma on subsequent generations, as it has proven to be more complex than previously thought. As a result, there has been specific focus on the parent/child relationship and how trauma specifically affects parenting in adult survivors of child abuse and neglect (Bottos and Nilsen, 2014). Research in this area has important implications in terms of how different service providers approach survivors of traumatic experiences in terms of parenting, attachment styles, and early childhood interventions, as childhood maltreatment is more likely to occur during essential moments of childhood development and its long-term effects can be especially adverse in these particular areas (Ford and Courtois, 2009).

Attachment theorists have posited that early relational experiences in childhood directly affects the organization of the attachment system, providing the working models on which later relationships will eventually be developed (Fonagy et al., 2002; Slade et al., 2005). In this context, exposure to childhood trauma, specifically trauma perpetrated by primary caretakers or under their care, can be detrimental to children's attachment (Carlson et al., 1989). Insensitive caregiving and maltreating behaviors have been implicated in the development of attachment insecurity and disorganization (van IJzendoorn and Bakermans-Kranenburg, 2009). In this context, we use the definition of trauma as defined by Bernstein et al. (2003) which includes physical abuse and/or neglect, emotional abuse and/or neglect, and sexual abuse. Children exposed to maltreatment have been found to be less likely to be securely attached, and more likely to fall under an insecure-disorganized attachment classification (van IJzendoorn and Bakermans-Kranenburg, 2009).

Exposure to insensitive, maltreating, or neglectful caretaking can affect a child's sense of safety and security, essential to the development of secure attachment (Cyr et al., 2010). In these contexts, a child can perceive their caretaker, their primary attachment figure, as a potential source of distress (Hesse and Main, 2006). As such, attachment working models have shown to remain generally constant over time, affecting attachment representations later on into adulthood.

Transitioning into parenthood is a period of reorganization of the self that carries with it inherent difficulties and can trigger memories and experiences associated to childhood (Lieberman and Van Horn, 2008; Fonagy, 2015). As a result, the attachment system is constantly activated resulting from different parent-child dynamics. The exposure to trauma in early childhood, namely abuse or neglect, has the potential to derail a parent's capacity to attend to their children, especially in moments of distress and has been found to impact their children's attachment style. Cues given by children when needing their caretakers can be misinterpreted by parents with a history of child abuse or neglect as threatening and overwhelming, and can lead to perceptual distortions that inhibit the child's needs from getting met and the parent's capacity to respond appropriately (Lieberman et al., 2015). This delineates the pioneering work by Fraiberg et al. (1975) that spoke to the “presence” of past traumatic experiences in the relational dynamics between caregiver and child. Finally, it is important to note that the experience of trauma alone does not necessarily lead to insecure attachment styles, however the experience of trauma that is not resolved or worked through has shown to lead to increased insecurity of attachment in children of parents who have experienced trauma early on (Berthelot et al., 2015).

## Parental reflective function

Parental reflective functioning is understood as the capacity to perceive and interpret human behavior in terms of intentional mental states, such as, needs, desires, goals, beliefs, etc., mentalization is imperative for healthy human development and more importantly, it is born out of the parent/child relationship (Fonagy et al., 1991, 2002; Ringel, 2011). Though human beings are born with the mechanisms that enable mentalization, it is a skill developed through mutual interactions between the child and his caregiver. Through parental modeling, children learn to identify their own affects and cognitions and those of others in order to communicate them and empathize appropriately (Fonagy et al., 2002). When this relationship becomes altered by the experience of traumatic events without the means to process and resolve the trauma, mentalization is one of the areas at risk of being compromised. Consequently, Lieberman et al. (2015) have written extensively on the repeated dynamic found in the clinical work concerning parents who have survived traumatic experiences and their tendency to experience their children as objects of transference, projecting onto them their own unmet needs from their childhood (Lieberman and Van Horn, 2008).

Child development that takes place in the context of maltreatment can be complex and varies in its effects, though mentalization is not compromised in all cases, there have been mentalizing problems associated to the experience of childhood trauma. Some of these include, difficulty understanding emotional expressions and social cues, less symbolic play, limited empathy for others, poor affect regulation, and difficulty identifying internal states (Ringel, 2011). Findings show that exposure to various traumatic experiences can lead to an absence of adequate parental mentalization of the child's experiences, ultimately affecting the way the child comes to understand himself and his surroundings (Fonagy, 1993). Seligman (2014) highlights that reflective thinking is important in order to feel secure in the world and find a sense of coherence. In contrast, when this is lacking, the world becomes a scary and unsafe place in which internal states cannot be made sense of or trusted and the behaviors of others become unpredictable and threatening. As a result, deficits in mentalization have been associated to labile self-organization, low self-esteem, isolation and fear, and in the worst of cases can be linked to psychopathology (Fonagy et al., 2002; Seligman, 2014).

The development of reflective functioning takes place within the context of a parent's own early attachment experience (Ensink et al., 2016). As a result, the experience of early childhood trauma can affect an adult's reflective functioning and in the transition to parenthood can potentially affect parental reflective functioning as well. Parental reflective functioning is defined as a parent's capacity to hold and reflect on their child's inner experiences, attributing mental states to behaviors and having the capacity to reflect on their own internal experience associated to their child's behaviors (Fonagy et al., 2002; Slade, 2008). Insofar as reflective functioning is developed in terms of the parent's developmental context, so too is parental reflective functioning, which is inherently connected to the parent's own experiences with their parental and attachment figures (Slade, 2008). As a result, parental experience of trauma is a potential factor influencing a parent's reflective functioning capacity. However, findings in this area have been mixed and suggest a more nuanced and complex relationship between early childhood trauma, parental reflective functioning, and attachment style (Schwerdtfeger and Nelson Goff, 2007). For example, a recent study by Ensink et al. (2014) indicates that the experience of childhood trauma alone doesn't necessarily compromise a parent's ability to respond to their child's attachment needs, rather, a parent's capacity to mentalize about their own trauma histories appears to significantly contribute to their capacity to tend to and satisfy their children's attachment needs and provide reflective caregiving. Ensink et al. (2014) have found that for parents who have experienced child abuse and neglect (CA&N), mentalization concerning trauma was associated to investment in pregnancy, positive feelings toward pregnancy, and quality of relationship to partner. This research suggests that for women who have experienced CA&N, the capacity to mentalize about traumatic events is particularly important, rather than mentalizing capacities in general (Berthelot et al., 2015). However, despite what research is still discovering on the mechanisms behind how trauma affects the mother/child relationship, the effects of trauma on maltreated children can potentially compromise the way they understand themselves and others, risking the development of mentalization (Cicchetti et al., 2003). This study specifically aims at taking a closer look at adult survivors of CA&N in terms of how parenting is influenced by the experience of childhood trauma and the potential effects on their offspring.

## Different types of trauma

Though trauma can be an all-encompassing construct that includes a spectrum of different experiences, we define trauma as abuse endured during childhood in terms of physical neglect or abuse, emotional neglect or abuse, and sexual abuse. These experiences all have the potential to have profound effects on a person, however it is important to understand the different effects that different types of trauma can have, as it is relevant both clinically and empirically. For example, child abuse reporting laws often define child abuse by the physical symptoms that a child can present, when symptoms associated to emotional trauma can be more complex and sometimes invisible, yet underreported or identified. Understanding the effects of different types of trauma can also shed light onto the intergenerational aspect of trauma when working with families who have extensive histories of trauma from one generation to another. A recent study by Bottos and Nilsen's (2014) was the first to take a close look at the relationship between distinct types of traumatic experiences and the experience of depression in adult survivors of abuse and its effect on maternal mentalization and the development of theory of mind in their offspring. Their findings highlighted the deleterious effects the experience of childhood trauma can have on survivors of CA&N and on their children. Bottos and Nilsen's (2014) study specifically highlighted the deleterious effects that emotional maltreatment had, in comparison to physical and sexual abuse. In their study, parental childhood experience of emotional maltreatment was found to be a significant predictor of children's mentalization outcomes. Lastly, another important contribution by Bottos and Nilsen's (2014) study is their finding regarding the interplay between depression, CA&N, reflective functioning, and theory of mind. They found that the experience of maternal depression in conjunction with emotional maltreatment had the most significant effects on reflective functioning and on their children's development of theory of mind. In summary, this particular study expands on an area of research that also interests the authors of this study in its differentiation between different types of traumatic experiences, as the experience of trauma can be very different from one person to another, and yet is often grouped into a single construct.

In this line, it seems plausible to hypothesize that emotional maltreatment has especially deleterious effects on maternal reflective functioning because of the very nature of emotional maltreatment and there have been some empirical findings that have backed up this hypothesis. For example, Rogosch et al. (1995) found that households characterized by emotionally labile caregiving proved to be unpredictable, thus affecting the child's ability to anticipate behaviors and assess consequences. Fear is also an important factor in households in which there is emotional maltreatment in that an environment colored by fear is not conducive to the expression on mental states and reflective capacities (Fonagy et al., 2002, 2007). In general, children who have experienced CA&N have been found to use less mental references, as they have been modeled in some cases not to do so and/or have been given messages that are at odds with their own experiences, leading to confusion, and distrust toward their internal states (Cloitre et al., 2006). This is especially alarming in light of the fact that emotional maltreatment is potentially one of the most underreported forms for maltreatment experienced by young children (Trickett et al., 2009). If this indeed are some of the experiences of children with the experience of CA&N, it leads to the question that is relevant for this study, which is what happens when these children are themselves parents?

## Current study

Following this theoretical line, this study seeks to contribute to the research focused on the survivors of trauma and its effects on parenting. While many studies have been focused on the immediate impact that trauma has during childhood, few studies have focused on the long term effects that childhood trauma has into adulthood and in parenthood, both in terms of adult attachment, and reflective functioning (RF; Ensink et al., 2014). This study seeks to describe the relationship that the experience of childhood trauma has on adult attachment and parental reflective functioning, as these three constructs are related and influence each other in various ways. Firstly, we hypothesize that insecure attachment will be related to higher prementalization, whereas secure attachment style will not. As a construct, reflective functioning was developed within the context of attachment theory. Studies on the relationship between RF and attachment style have found the two to be intricately related, parental RF has not been the exception. Through the use of the Adult Attachment Interview, studies have found that parents who rated high on RF tend to be securely attached adults and have children who are securely attached as well (Slade, 2005). In fact, it is thought that the intergenerational transmission of attachment style is transmitted through the parent's RF capacity (Fonagy et al., 2002). Lower PRF will be measured by levels of prementalization, where higher prementalization is indicative of lower PRF and vice versa. Our second hypothesis is that emotional abuse and emotional negligence will yield higher levels of insecure attachment style and prementalization in comparison to the other types of trauma assessed (physical abuse, physical neglect, and sexual abuse). This hypothesis derives from recent literature that highlights that emotional abuse is an underlying factor at the root of other forms of abuse (Bottos and Nilsen, 2014). While all forms of abuse are aggressions toward the well-being of children, trauma-related literature has highlighted that emotional abuse during childhood can deeply fragment an individual's sense of self, attachment representations, and mentalizing capacities (Bottos and Nilsen, 2014). Emotionally abusive and negligent contexts deprive children of adults that help them make sense of, reflect on, and create a coherent narrative of life events, especially traumatic ones, ultimately compromising their own RF abilities (Ensink et al., 2014).

Recent studies suggest a more nuanced profile of adults with a history of childhood trauma, both in terms of attachment classification and RF capacities. For example, Ensink et al. (2014) and Stovall-McClough et al. (2008) both found that in a non-clinical sample of women a significant number of participants with the experience of child abuse and neglect had secure attachment suggesting that more research is needed in this area, especially studies that include non-clinical samples in order to illuminate the nuanced effects that childhood traumatic experience have into adulthood.

## Methods

### Participants

The participants for this study belong to secondary data from the FONDECYT 1130786 project which took place in Santiago, Chile. There were 125 participants who were evaluated during the year 2014–2015 in daycare centers belonging to JUNJI. Generally, parents and children who attend JUNJI daycare centers belong to a medium-low socioeconomic stratum of Chilean society. There is a high number of single mothers within this population and many of the children who participated are under the care of other family members like grandmothers/grandfathers, uncles/aunts, cousins, etc. One-hundred and 56 participants were initially recruited, but only 124 participants finished the battery of assessment.

### Procedure

As previously mentioned, the data obtained for this study is secondary data belonging to the FONDECYT project #1130786. In order to obtain this data contact was initially established with JUNJI centers after they were presented with the study in which they were invited to participate after signing the appropriate consent forms. Once the consent forms were obtained from the director of the centers, parents and caregivers were invited to participate after signing the appropriate consent forms as well. Later, a sociodemographic questionnaire was given to the parents and caregivers to complete, followed by the other measures. The children who participated in the FONDECYT project #1130786 also filled out questionnaires, but for the purposes of this study, these assessments were not incorporated. The procedures included all comply with the requirements of the Ethics Committee of the Universidad Católica, Chile.

### Measures

#### Maternal experience of childhood trauma

To assess for maternal trauma during childhood, the participants were asked to complete the Child Trauma Questionnaire (CTQ) developed by Bernstein et al. (2003). The CTQ assesses for past experiences of trauma and specifically measures physical abuse, physical neglect, emotional abuse, emotional neglect, and sexual abuse. The CTQ is a self-report questionnaire that measures 5 categories of childhood trauma experience, including emotional, physical, and sexual abuse as well as emotional and physical neglect. Each subscale is measured in 5 items rated on a 5-point Likert scale from 1 (never true) through 5 (very often true).

#### Experiences in close relationships (ECR)-adult attachment (Brennan et al., 1998)

This instrument measures the individual's attachment style in the context of romantic partnership. This instrument allows us to measure adult attachment on a continuum focusing on two dimensions: avoidance (inconformity with intimacy and dependency) and anxiety (fear of separation and abandonment). It is composed of 36 items, measured on a Likert scale with a possible score from 1 to 7 (1 = completely disagree and 7 = completely agree). Scores indicate 4 types of attachment styles: Secure (low avoidance, low anxiety), Preoccupied (low avoidance, high anxiety), disengaged (high avoidance, low anxiety), and fearful (high avoidance, high anxiety). Both dimensions have confidence indictors of 0.94 and 0.91 via Cronbach's Alpha. Chilean studies using this measure show a reliability of 0.87 for avoidance and 0.85 for anxiety (Rivera, 2006). This study used the version of the ECR validated in Chile in 2012 by Spencer, Guzman, Fresno, & Ramos which consists of 12 items and has a adequate reliability (Alpha's Secure = 0.64 / Alpha's unsecure = 0.78).

#### Parental reflective function

The Parental Reflective Functioning Questionnaire- PRFQ (2009) was created by Luyten, Mayes, Sadler, Fonagy, Nicholis, Crowley, Vesper, Mobley, Stewart, Close, & Slade. This is a 39-item, self-report measure which were translated in Mexico with a inter-judge verification conducted in Chile. It consists of three subscales which measure the caregiver's curiosity about their child's mental states and how they relate mental states to behavior, resulting in a measure of low, medium, or high parental reflective functioning. Each item in the PRFQ are given up to 7 points in a Likert type scale. On this scale 1 point indicates complete disagreement and 7 points indicate complete agreement. The prementalization scale consists of non-mentalizing items. The mental states subscale consists of items evaluating the inability to recognize mental states that are not transparent. The third subscale was designed to measure interest and curiosity in the parent regarding his/her child's mental states.

#### Sociodemographic characteristics

Sociodemographic characteristics were taken through a questionnaire completed by participants. The questionnaire included items related to maternal age, child's age, and maternal educational level.

**Sociodemographic characteristics**.

**Table:** 

**Variable**	***n***	**Mean**	**Std. Dev**.	**Min**	**Max**
Child's age	124	44.65323	3.737406	36	54
Mother's age	124	29.68551071	6.551071	19	47
**Child_Sex**		**Frequency**	**Percent**	**Cum**.	
Male		62	50.41	50.41	
Female		61	49.59	100

**Table:** 

**Maternal educational level**		**Frequency**	**Percent**	**Cum**.
No response		2	1.61	1.61
Did not complete middle school		4	3.23	4.84
Completed middle school		8	6.45	11.29
Did not complete high school		19	15.32	26.61
Acquired high school degree		55	44.35	70.97
Did not complete college		27	21.77	92.74
Acquired college degree		8	6.45	99.19
		1	0.81	100

## Data analyses

The analyses were conducted using regression models in order to estimate the lineal relationship among variables, furthermore, the non-linear relationships were estimated using interaction models. The Stata software was used to carry out the descriptive correlational and regression models. Finally, in order to graphically show the interaction effects, we used marginal estimation Stata 14 module.

The equations used were the following:

(1) $$\begin{array}{r} \left(Prem\right)\;=a+b1\text{\_}\left(Ins.Attach\right)+\;b2\text{\_}\left(Sec.\;Attach\right) \end{array}$$

(2) $$\begin{array}{r} \left(Prem\right)\;=a+b1\text{\_}\left(P.\;Abandonment\right) \end{array}$$

(3) $$\begin{array}{r} \left(Prem\right)\;=a+b1\text{\_}\left(E.\;Abandonment\right) \end{array}$$

(4) $$\begin{array}{r} \left(Prem\right)\;=a+b1\text{\_}\left(P.\;Negligence\right) \end{array}$$

(5) $$\begin{array}{r} \left(Prem\right)\;=a+b6\text{\_}\left(E.\;Negligence\right) \end{array}$$

(6) $$\begin{array}{r} \left(Prem\right)\;=a+b1\text{\_}\left(Ins.Attach\right)+\;b2\text{\_}\left(Sec.\;Attach\right)+\\ b3\text{\_}\left(P.\;Abandonment\right)+b4\text{\_}\left(E.Abandonment\right)+\\ b5\text{\_}\left(P.\;Negligence\right)+b6\text{\_}\left(E.\;Negligence\right) \end{array}$$

(7) $$\begin{array}{r} \left(Prem\right)\;=a+b1\text{\_}\left(Ins.Attach\right)+\;b2\text{\_}\left(Sec.\;Attach\right)+\\ b3\text{\_}\left(P.\;Abandonment\right)+b4\text{\_}\left(E.Abandonment\right)+\\ b5\text{\_}\left(P.\;Negligence\right)+b6\text{\_}\left(E.\;Negligence\right)+\\ b7\text{\_}\left(Ins.Attach*P.Negligence\right) \end{array}$$

## Results

The results are presented in the following three sections. Firstly, we report the descriptive statistics and correlations for all measures included in the model. Second, we show seven regression models in order to test the hypotheses presented in this paper. Third, we show a seventh model that estimates the moderation role of physical negligence.

From the results presented in Table 1, it can be inferred that the variables considered in the model exhibited reasonable levels of variability. All of the correlations indexes were, as expected, positively related to prementalization (except in the case of secure autonomous attachment style) and all means were significantly different from zero.

**Table 1:** **Correlation among variables**.

		**1**	**2**	**3**	**4**	**5**	**6**	**7**
1	Pre-mentalisation							
2	Insecure attachment	0.312^**^						
3	Secure autonomous attachment	−0.109	0.100					
4	Physical abandonment	0.050	0.148	0.033				
5	Emotional abandonment	0.035	0.273^**^	−0.034	0.590^**^			
6	Physical negligence	0.048	0.224^*^	−0.114	0.315^**^	0.491^**^		
7	Emotional negligence	0.160^+^	0.238^*^	−0.228^*^	0.309^**^	0.418^**^	0.624^**^	
	Mean	2.516	3.006	4.940	1.453	1.789	1.486	1.939
	Estandar deviation	1.216	1.337	1.402	0.775	0.949	0.541	0.932

** *p < 0.01*,

* *p < 0.05*,

+ *p < 0.10*.

Table 2 shows the results of the regression models estimating the hypotheses. Model 1 shows that only insecure attachment is related to the adult prementalization scores. This specific result indicates that adults with insecure early attachment show higher inabilities to recognize the mental states of their children (higher prementalization). On the other hand, secure attachment did not show this effect. Models 2, 3, 4, and 5 shows that any kind of negligence or abandonment was related with prementalization. Model 6 estimates the joint effect of variables. In this model, controlling for negligence and abandonment yielded a positive effect in terms insecure attachment, which is a similar effect to the one observed in model 1. Up to this point, results indicate that the unique relevant dimension is attachment, particularly insecure attachment style.

**Table 2:** **Regression model**.

	**(1)**	**(2)**	**(3)**	**(4)**	**(5)**	**(6)**	**(7)**	**(8)**
Insecure attachment	0.30^**^					0.30^**^	−0.20	−0.20
	(3.78)					(3.66)	(−0.41)	(−0.83)
Secure autonomous attachment	−0.12					−0.11	−0.11	−0.11
	(−1.70)					(−1.41)	(−0.16)	(−1.44)
Physical abandonment		0.08				0.08	0.05	0.05
		(0.56)				(0.62)	(0.75)	(0.33)
Emotional abandonment			0.05			−0.13	−0.10	−0.10
			(0.39)			(−0.38)	(−0.52)	(−0.62)
Physical negligence				0.11		−0.21	−1.26^*^	−1.26^**^
				(0.54)		(−0.43)	(−2.31)	(−2.63)
Emotional negligence					0.21	0.18	0.18	0.18
					(1.80)	(0.24)	(1.21)	(1.41)
Insecure x physical negligence							0.34^*^	0.34^*^
							(2.19)	(2.37)
Constant	2.23^**^	2.40^**^	2.44^**^	2.35^**^	2.11^**^	2.21^**^	3.65^**^	3.74^**^
	(5.20)	(10.34)	(10.43)	(7.36)	(8.46)	(4.12)	(4.14)	(4.18)
Observations	125	125	125	125	125	125	125	125
R-squared	0.116	0.003	0.001	0.002	0.026	0.135	0.164	0.168
*F*-test (*p*-values)	0.00	0.58	0.70	0.59	0.07	0.01	0.00	0.00

t-statistics in parentheses

** *p < 0.01*,

* *p < 0.05*.

Model 7 shows the moderation effect of physical negligence in the relationship between insecure attachment and prementalization. The general result indicates that the strength of relationship between both variables changes depending on the specific type of negligence. Particularly, when the declared physical negligence is zero the effect of insecure attachment on prementalization was non-significantly different from zero (*0.20, p* = *0.409*). However, when the declared physical negligence is higher (1) the effect of insecure attachment on mentalization is positive *(*−*0.20* + *0.34* = *0.14, p* = *0.030*).

In order to check the collinearity, the variance inflation factor (VIF) was estimated. The average of VIF was 1.55 indicating that the independent variables are not collinear. Furthermore, normality was estimated using Shapiro Wilks test, which indicates that the residuals are non-normal (W = 0.944; *p* < 0.01). This indicates that these results must be interpreted considering this assumption's violation. Nevertheless, it is not possible to observe severe outliers, indicating that the non-normality is not a severe violation. Additionally, in order to handle potential effects of outliers, a robust regression was estimated (model 8). Results indicate that the observed effects are robust.

**Figure 1:**
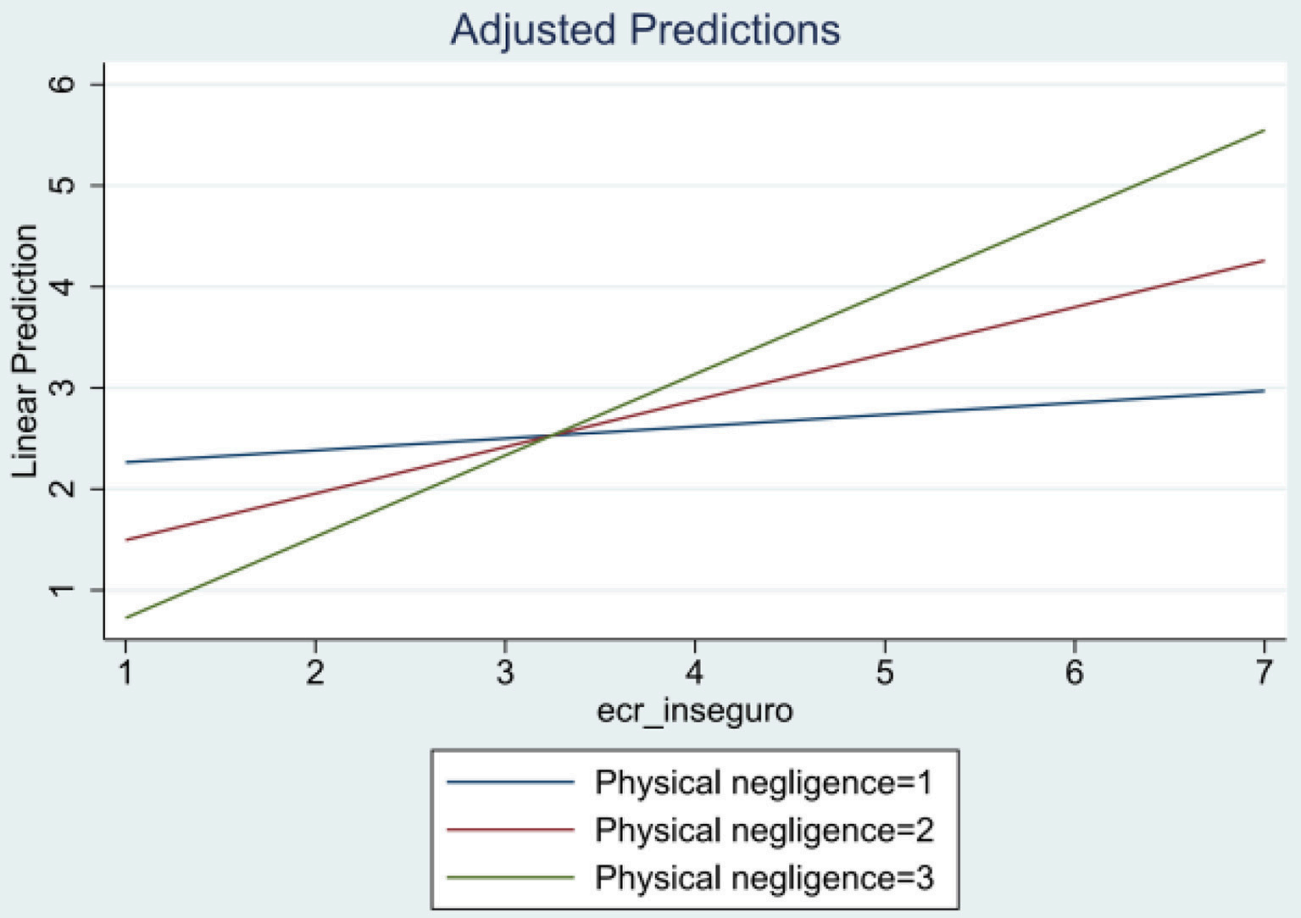
**Moderation effect between physical negligence and insecure attachment**.

Figure 1 shows a graphical representation of the moderation effect. On the one hand, the marginal estimation indicates that at lower levels of physical negligence the fitted line is almost flat. This means that when participants did not report physical negligence, prementalization is almost the same at different levels of insecure attachment. While on the other, when participants reported higher levels of physical negligence, the inability to recognize the mental states of their children varied significantly in the different levels of insecure attachment. For example, a participant that reported 3 points in physical negligence showed significantly lower levels of prementalization at level 1 of insecure attachment and showed very high levels of prementalization at level 7 of insecure attachment.

## Discussion

The results of this study yield interesting implications. Firstly, we see that maternal experience of physical abandonment, emotional abandonment, physical negligence, and emotional negligence were significantly correlated amongst each other. This is congruent with the literature regarding the experience of trauma which has shown that one form of maltreatment often coexists with multiple other forms of maltreatment (Trickett et al., 2009). As Bernstein et al. (2003) point out, it is often difficult to disentangle one form of trauma from others and due to the nature of trauma, it can be challenging to adequately capture the trauma phenonemnon in the assessment process, particularly when it comes to differentiating abuse from neglect. However, the CTQ-SF, yields considerable discriminate validity between the constructs even though they are highly interrelated (Bernstein et al., 2003).

The next set of results found in this study pertain to the relationship found between insecure attachment and the prementalization scale. Healthy reflective functioning has been found time and time again to be born out of relationships characterized by secure attachment (Fonagy et al., 2002; Allen et al., 2008). Securely attached parent/child dyads promote the development of mentalizing capacities from infancy onward and allow the child opportunities to learn and deepen his/her understanding about mental states and eventually apply this knowledge within their own social circles (Luyten et al., 2009). Furthermore, attachment style and reflective functioning has been found to be intricately linked in that we have come to understand reflective functionig as the means throught which attachment patterns are passed on intergenerationally (Fonagy et al., 1991). In this particular study, the correlation between the two is evidenced by the regression model in which we found that insecure attachment predicted the likelihood of the parent scoring highly in the prementalization scale of the PRFQ. This subscale consists of items composed of non-mentalizing examples, which is further evidence of that insecure attachment styles in adults are related to their inability to mentalize adequately when it comes to those around them. Previous studies have also confirmed these results, for example, Fonagy et al. (1991) and Slade (2005) both found that secure attachment classification through the Adult Attachment Interview has higher RF scores than their securely attached counterparts.

As a result of a regression modeling we conclude that the presence of insecure attachment and the experience of physical negligence showed the most damaging effects on reflective functioning. This can be found in some of the existing literature regarding maltreated children who as a result of traumatic experiences of abuse and neglect with their caregivers, experience significant disruption in their mentalizing abilities, as these households are characterized by unresponsive, unavailable, and parental detachment from the child's needs and rights. Rogosch et al. (1995) pose that households which are characterized by maltreatment convey a lack of RF and can be perceived as an explicit negation of the child's internal experience which leads to confusion in the child between what they experience internally and how his/her caregivers respond. This ultimately affects their core sense of self and it becomes increasingly more difficult to understand their own mental states and the mental states and desires of those around them, making it difficult to assess and predict social behaviors of peers and counterparts. Our findings also show that insecure attachment did not appear to affect prementalization if there was an absence of physical negligence. However, in the presence of physical negligence, insecure attachment significantly worsens mentalization capacity (as seen in the increase of prementalization scores). Interestingly, this relationship was not present in the other dimensions of trauma captured by this particular instrument. In summary, only the presence of significant physical negligence in the mother's early childhood experience impacted the extent to which maternal insecure attachment worsened mentalization capacities.

As we see in Table 2, lower scores in the physical negligence do not alter the relationship between insecure attachment and prementalization, however, as the physical negligence scores increase, there is a significant increase in the prementalization scores, which leads us to conclude from these results that the mothers who present insecure attachment and report the experience of physical neglect in their childhood, were significantly and negatively affected in their reflective functioning. Its seems from these results that the exposure to physical negligence had a particular effect. Physical negligence seems to play a role in the experience of trauma that is commonly associated to other types of traumas and consequences thereafter. For example, in a national study conducted in the United States characterizing adults who experienced physical abuse during childhood found that neglect was significantly reported in addition to other types of trauma and those who experience neglect showed significantly lower perceived parental support as a child (Sugaya et al., 2012), this is consistent with what the CTQ was meant to measure in terms of physical neglect in that this construct refers to the child's parent's inability to provide appropriate parental supervision, putting in jeopardy the child's safety and well-being (Bernstein et al., 2003). Studies have found that abusive and neglectful households are often characterized by emotional lability and chronic stress stemming from being unable to predict accurately what will trigger their abuser (Sugaya et al., 2012; Bottos and Nilsen, 2014). The results of our study fall in line with the characterization of physically negligent households in that growing up in this context disturbs key developmental stages, particularly when it comes to mentalization. Physically abusive and negligent households make it especially difficult for children to predict and assess the antecedents and consequences of behavior, a key element in the development of mentalization (Rogosch et al., 1995). Physical negligence is also an overt negation of the developing child's internal experience in that these painful experiences are often refuted by the parental figure, making it increasingly difficult for the child to develop an internal dialogue through which to process the trauma, layered with the lack of space to communicate about internal states with parental figures. In a study focused on the effects of early neglect on children, Spratt et al. (2012) found that of physical abuse, sexual abuse, emotional abuse, and physical or medical neglect, adults who reported neglect in their childhood showed the strongest association with delays relating to overall language development which depraved them of healthy language, cognitive, and behavioral development; these children specifically showed significant social and interpersonal impairment, as they were not able to effectively communicate, affecting their overall social interactions. Our study sought to focus on the long-term effects of this kind of abuse by examining its effect on mentalization. Our findings fall in line with findings by Bottos and Nilsen (2014) in that an often-underreported experience of trauma, in our study physical negligence and in their study, emotional maltreatment, was found to have significant effects on adult's qualities related to caregiving. Our findings highlight the nuanced and complex effects that these early experiences have on survivors of childhood trauma, however more research is needed in order to begin to understand the full effects of childhood trauma through the lifespan.

## Limitations

As has been previously noted by other authors who have conducted studies on the experience of past trauma, it can be a challenge to ask a survivor of trauma to accurately convey memories of their painful experiences (Bernstein et al., 2003). In light of these difficulties, retrospective assessments can yield important contributions to the work of understanding trauma in its various contexts and manifestations. Though minimizations, normalization, and, in some cases, dissociation, can play a role in the self-report method of assessing trauma, the CTQ-SF incorporates a minimizing/deniability scale developed by the authors of the instrument to detect underreporting.

## Ethics statement

Pontificia Universidad Católica, Escuela de Psicología, Comité de Etica. The parents who participated in this study were given a consent form which detailed the type of assessment they would partake in and in which they were invited to be part of a 5 week group focused on strengthening the attachment bond and mentalizing capacities between the parent and child. The consent form explicitly stated that their participation was voluntary, that their information was confidential, and that they were able to refuse participation at any time during during the study without consequence. Parents were asked to sign the consent form after reading it in detail. The preschools to which the participants belonged were also asked to sign a consent form acknowledging that they agreed with the participation of their parents. Parents were also informed that the data resulting from their initial assessment were confidential and only used for educational and research purposes. Minors only participated with the consent of their caregivers and in the presence of their caregivers.

## Author contribution

PS: conception and design of work; interpretation of data; drafting of article. MS: data collection; critical revision of article. DM: data analysis, critical revision of article.

### Conflict of interest statement

The authors declare that the research was conducted in the absence of any commercial or financial relationships that could be construed as a potential conflict of interest.
